# Unintentional injury mortality in India, 2005: Nationally representative mortality survey of 1.1 million homes

**DOI:** 10.1186/1471-2458-12-487

**Published:** 2012-06-28

**Authors:** Jagnoor Jagnoor, Wilson Suraweera, Lisa Keay, Rebecca Q Ivers, JS Thakur, Prabhat Jha

**Affiliations:** 1 Centre for Global Health Research, Li Ka Shing Knowledge Institute, St. Michael’s Hospital, and Dalla Lana School of Public Health, University of Toronto, Toronto, Canada; 2 The George Institute for Global Health and Sydney School of Public Health, The University of Sydney, Sydney, Australia; 3 School of Public Health, Post Graduate Institute of Medical Education and Research, Chandigarh, India; 4 World Health Organisation, Country Office, New Delhi, India

**Keywords:** Unintentional-injuries, Mortality, Verbal autopsy, India

## Abstract

**Background:**

Unintentional injuries are an important cause of death in India. However, no reliable nationally representative estimates of unintentional injury deaths are available. Thus, we examined unintentional injury deaths in a nationally representative mortality survey.

**Methods:**

Trained field staff interviewed a living relative of those who had died during 2001-03. The verbal autopsy reports were sent to two of the130 trained physicians, who independently assigned an ICD-10 code to each death. Discrepancies were resolved through reconciliation and adjudication. Proportionate cause specific mortality was used to produce national unintentional injury mortality estimates based on United Nations population and death estimates.

**Results:**

In 2005, unintentional injury caused 648 000 deaths (7% of all deaths; 58/100 000 population). Unintentional injury mortality rates were higher among males than females, and in rural versus urban areas. Road traffic injuries (185 000 deaths; 29% of all unintentional injury deaths), falls (160 000 deaths, 25%) and drowning (73 000 deaths, 11%) were the three leading causes of unintentional injury mortality, with fire-related injury causing 5% of these deaths. The highest unintentional mortality rates were in those aged 70years or older (410/100 000).

**Conclusions:**

These direct estimates of unintentional injury deaths in India (0.6 million) are lower than WHO indirect estimates (0.8 million), but double the estimates which rely on police reports (0.3 million). Importantly, they revise upward the mortality due to falls, particularly in the elderly, and revise downward mortality due to fires. Ongoing monitoring of injury mortality will enable development of evidence based injury prevention programs.

## Background

Indirect estimates by the World Health Organization (WHO) and the Global Burden of Diseases Study (GBD) suggest that unintentional injuries account for 3.9 million deaths worldwide [1], of which about 90% occur in low- and middle-income countries. The majority of these deaths are attributable to road traffic injuries, falls, drowning, poisoning and burns [1].

In 2004, WHO estimated about 0.8 million deaths in India were due to unintentional injuries [1]. Direct Indian estimates of unintentional injury deaths relying on annual National Crime Records Bureau (NCRB) reports of injury deaths from police records showed only 0.3 million injury deaths in 2005 [2], but police record are subject to under-reporting and misclassification [3-5]. Other sources of mortality data from selected health centres in rural areas [6], and selected urban hospitals [7] are not representative of the population of India, and have other methodological limitations [8].

The objective of this paper is to estimate total unintentional injury mortality in India and its variation by gender, rural/urban residence and region using results from a nationally representative survey of the causes of deaths.

## Methods

### Study setting and data collection

The Registrar General of India (RGI) randomly selected 6671 small areas from approximately one million small areas defined in the 1991 census for its Sample Registration System (SRS) [9]. In 1993, household characteristics of the SRS areas, each with about 150 houses and 1000 people, were documented. The SRS sample frame covered 6.3 million people in all 28 states and seven union territories of India. SRS sampling frame is based on the results of census of India, which is conducted every ten years. The selected households are continuously monitored for vital events by two independent surveyors. The first is a part-time enumerator (commonly a resident of the village/area or a local school teacher familiar with the area/village) who visits the households every month. The second is a full-time (nonmedical) Registrar General of India’s surveyor who visits the households every 6 months. Another staff member from the office of Registrar General of India does the reconciliation of vital events reported by the part-time enumerator and the full-time surveyor, arriving at a final list of births and deaths for each household, at the completion each half-yearly survey.

In the last decade, the RGI has introduced an advanced form of verbal autopsy called “RHIME” (Routine, Reliable, Representative and Re-sampled Household Investigation of Mortality with Medical Evaluation) [9,10]. Verbal autopsy is a method of ascertaining the cause of death by seeking information on signs, symptoms and circumstances from a family member or care taker of the deceased [10]. Since 2001, about 800 non-medical graduates who were full time employees of the RGI, had knowledge of local languages and were trained to implement the RHIME method visited the families to record events preceding each death using three age specific questionnaires (neonatal, child and adult) including a narrative in the local language. The neonatal (0–28 days) and child death (29 days–14 years) questionnaires included a direct question, “Did s/he die from an injury or accident? If yes, what was the kind of injury or accident?” Response options included 1) Road traffic accident 2) Falls 3) Fall of objects (on to the person) 4) Burns 5) Drowning 6) Poisoning 7) Bite/sting 8) Natural disaster 9) Homicide/assault 10) Unknown. Place of death was recorded in all deaths with response options of 1) Home 2) Health facility (like government hospitals, private hospitals and registered practitioners) and 3) Others (including road side, public area, on transportation and body of water). A random sample of about 5% of the areas was resurveyed independently generally with consistent results. Details of the methods, validation results, and preliminary results for various diseases have been reported previously [10-14].

### Cause of death assignment

The field reports including the individual narratives were sent randomly based on the language, to at least two of 130 physicians who were specially trained in disease coding. The physicians assessed the underlying cause of death and assigned a three character code from the International Classification of disease (ICD), 10^th^ Revision [15]. Unintentional injury deaths were allocated ICD codes from chapter XX for external causes of morbidity and mortality, including V01-X59, Y40-Y86, Y88, and Y89 codes. In case of chapter level disagreement between the two physicians, the final ICD code was assigned by a third senior physician. In case of sub chapter disagreements, specific codes were adjudicated by a specific codes were adjudicated two members of the research team.

Reports could not be collected for 12% of the identified deaths mostly due to migration of the household or change of residence; this is unlikely to have led to any systematic misclassification in cause of death as these missing deaths were distributed across all states.

Moreover, the SRS definition of usual resident included those who travel away from home for periodic work [9], so deaths away from home were captured provided the whole household had not moved. A total of about 136,480 deaths were identified between January 1, 2001 and December 31, 2003. About 9% of all the death reports could not be coded due to field problems such as poor image quality of the narrative or insufficient information; hence cause of death was identified for 122,828 deaths.

### National estimates for absolute number of deaths and mortality rates

We applied the proportion of each cause of death to the UN Population Division estimates of Indian deaths from all causes in 2005 ((9.8 million; upper and lower limits 9.4 and 10.3 million respectively) [13,16]. UN estimates were used for more accurate calculation of deaths and mortality rates (using Preston and Coale method) [17] because the SRS undercounts mortality by approximately 10% [18,19]. All major cause of death like malaria, HIV and child mortality [12,13] has been estimated for the year 2005 from the Million Death Study making cause specific mortality comparable for policy implications.

The proportion of cause specific deaths in each age category was weighted to the SRS sampling fractions in the rural and urban parts of each state. However, unweighted proportions yielded nearly identical results [20]. Application of the data from 2001–03 to 2005 deaths should not introduce major bias as there was little change in the yearly distribution of cause of death in the present study (p = 0.736 for yearly variation in proportional mortality). Sub national estimates were produced for six major regions (north, south, west, central, northeast, and east) [9] from segregating the national UN totals by the relative SRS death rates, as described earlier [21]. Live births totals from UN were used to calculate mortality rates for children younger than five years [21,22]. Confidence interval (95%) for each cause of death proportion or mortality rate was calculated (using the Taylor linearization method) on the basis of the survey design and the observed sample deaths in the present study [23].

SRS enrolment is on a voluntary basis, and its confidentiality and consent procedures are defined as part of the Registration of Births and Deaths Act, 1969. Oral consent was obtained in the first SRS sample frame. Families are free to withdraw from the study, but the compliance is close to 100%. The study poses no or minimal risks to enrolled subjects. All personal identifiers present in the raw data are anonymised before analysis. The study has been approved by the review boards of the Post-Graduate Institute of Medical Education and Research, St. Michael’s Hospital and the Indian Council of Medical Research.

## Results

Unintentional injuries accounted for 7% (8023/122,828) of all deaths (Table 1). A small number (155; 1.9%) of injury deaths were excluded from analyses as intent (unintentional or intentional) could not be determined. Unintentional injury deaths constituted nearly 20% of total deaths at ages 5–29 years and 12% of total deaths at ages 30–44 years. Over 80% (6621) of unintentional injury deaths occurred in rural areas. More males (5228) than females (2795) died from unintentional injury, and male deaths exceeded female deaths at all ages except beyond 70 years.

**Table 1:** Unintentional injury attributed deaths in the Sample Registration System 2001–2003 and estimated national rates for 2005 by age, sex and place of residence

**Age range in years**	**Sample Registration System deaths, 2001-03**				**All India, 2005 Unintentional injury rate per 100 000**								
	**Number of unintentional injury deaths**				**Rural**			**Urban**			**National**		
	**Male**	**Female**	**Total**	**% of all cause deaths**	**Male (95% CI)**	**Female (95% CI)**	**Total (95% CI)**	**Male (95% CI)**	**Female (95% CI)**	**Total (95% CI)**	**Male (95% CI)**	**Female (95% CI)**	**Total (95% CI)**
0–4*	410	366	776	3	3.6 (3.2,4)	3.1 (2.8,3.4)	3.4 (3.1,3.7)	1.7 (1.1,2)	1.7 (1.2,2.2)	1.7 (1.3,2.1)	3.0 (2.7,3.3)	3.0 (2.7,3.3)	**3.0 (2.8,3.2)**
5–14	459	272	731	19	36 (32,39)	23 (20,26)	30 (27,32)	17 (12,22)	17 (13,29)	17 (13,21)	31 (28,34)	22 (20,24)	**27 (25,29)**
15–29	1207	423	1630	18	59 (55,62)	24 (21,26)	42 (40,44)	49 (42,55)	18 (14,23)	34 (30,38)	56 (52,59)	22 (20,24)	**40 (37,41)**
30–44	1042	290	1331	12	78 (73,84)	25 (21,28)	52 (49,55)	64 (55,72)	18 (13,23)	43 (38,48)	74 (69,78)	23 (20,26)	**50 (47,52)**
45–59	832	288	1120	6	94 (87,101)	39 (33,43)	67 (62,71)	77 (65,89)	35 (26,44)	58 (50,65)	88 (82,94)	38 (33,42)	**64 (60,67)**
60–69	478	352	829	4	151 (136,166)	115 (102,128)	133 (123,142)	136 (107,165)	84 (62,106)	110 (92,128)	151 (138,165)	107 (96,119)	**128 (120,137)**
70 +	800	803	1603	5	431 (397,463)	371 (341,400)	399 (377,421)	408 (342,475)	464 (398,530)	439 (392,485)	421 (392,450)	399 (371,426)	**410 (384,429)**
**All ages (% or 95% CI)**	**5228 (65)**	**2795 (35)**	**8023 (100)**	**7**	**75 (73,77)**	**44 (43,46)**	**60 (59,62)**	**60 (57,63)**	**39 (35,42)**	**50 (47,52)**	**72 (70,74)**	**43 (42,45)**	**58 (56,59)**

*Mortality rate per 1000 live births.

The national mortality rate (MR) for unintentional injury per 100 000 population was estimated to be 58 (males 71, females 43), with higher rates in rural (60) than urban areas (50). The mortality rates were highest at ages 70 years or higher (410/100 000), with falls accounting for 63% of all injury deaths in this age group. In absolute terms, during 2005, about 648 000 deaths from unintentional injuries occurred in India (95% CI 634 000-662 000; Table 2).

**Table 2:** Number of unintentional injury deaths by type, in the Sample Registration System, 2001–2003 and estimated national totals for 2005

**Unintentional injury type (ICD codes)**	**Number of Sample Registration System deaths 2001–03 (n)**	**Estimated national deaths, 2005 (1000’s)**	**Standardized mortality rate per 100 000 population, 2005**		
		**(95% CI)**	**Male**	**Female**	**Total**
			**(95% CI)**	**(95% CI)**	**(95% CI)**
**Road traffic injuries** (V01-V89,V99)	2339	185 (178,193)	26.3 (25.2,27.5)	6.0 (5.4,6.5)	**16.5 (15.7,17.4)**
**Falls** (W00-W19)	2003	160 (153,167)	14.9 (14.0,15.8)	14.2 (13.3,15.1)	**14.3 (13.5,15 .2)**
**Drowning** (W65-W74)	903	73 (68,77)	8.2 (7.6,8.9)	4.6 (4.0,5.1)	**6.4 (5.9,6.9)**
**Contact with venomous plants and animals** (X20-X29)	643	53 (49,57)	5.0 (4.5,5.5)	4.3 (3.8,4.8)	**4.7 (4.2,5.2)**
**Mechanical forces** (W20-W64)	459	38 (34,41)	4.4 (4.0,4.9)	2.2 (1.9,2.6)	**3.4 (3.0,3.8)**
**Fires** (X00-X09)	375	34 (30,37)	1.8 (1.5,2.2)	4.2 (3.7,4.8)	**3 (2.6,3.4)**
**Forces of Nature** (X30-X39)	380	33 (29,36)	3.2 (2.8,3.7)	2.4 (2.1,2.8)	**2.8 (2.5,3.2)**
Other unintentional injuries* (V90-V98, W75-W84, X10-X19, X50-X59, W85-W99, X40-X49, Y40-Y86, Y88)	918	73 (68,78)	7.5 (7.1,8.2)	5.2 (4.6,5.7)	**6.3 (5.8,6.8)**
**Unintentional injuries (V01-X59, Y40-Y86, Y88, Y89)**	**8023**	**648 (634,662)**	**71.9 (69.9,73.8)**	**43.1 (41.5,44.7)**	**57.9 (56.7,59.2)**

*Other injuries include transport accidents other than RTI, poisoning, exposure to electric current, radiation, extreme ambient air temperature and pressure, contact with hot substances, other accidental threat to breathing, overexertion, travel and privation and adverse of medical and surgical interventions. Details of ICD codes are available from *http://apps.who.int/classifications/icd10/browse/2010/en#/XX.*

^†^ Deaths due to undetermined intent (Y10- Y34) were 155 (2% of all injury deaths); of these 15% of these were Y14 (Poisoning by exposure to unspecified drugs, medicaments and biological substance, undetermined intent).

Road traffic injuries (RTI) were the leading cause of death, accounting for 185 000 deaths or 29% of all unintentional injury deaths (MR = 16.5), followed by falls (160 000, 25%; MR = 14**.**3) and drowning (73 000; 11%; MR = 6.4). Males had higher mortality rates for all sub-types of unintentional injuries except for fire-related deaths. Females aged 15–29 years (9,900; MR = 5.8, 95% CI 4.9- 6.4) had the highest mortality rates from fire. The ratio of male to female mortality rates were as follows: RTI (4:1), drowning (2:1); fires (1:3); and falls (1:1).

**Figure 1:**
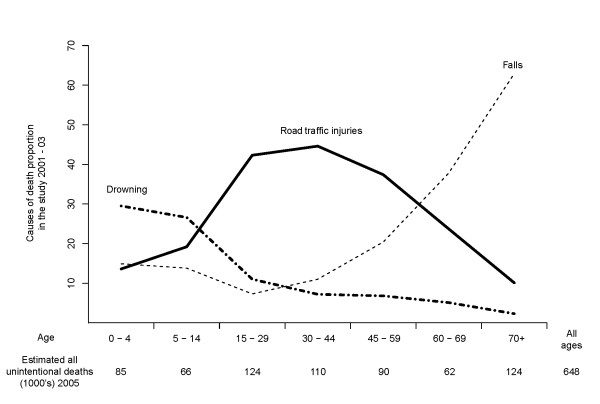
**Age-distribution of unintentional injury mortality for the three leading causes of injuries in India, 2005.** The three leading causes of unintentional injuries are presented as a proportion of all unintentional deaths in the sample.

Figure 1 provides the age distribution for the top three causes of unintentional injury deaths. RTI were the leading cause of death at ages 15–59 years (41% of all unintentional injury deaths in the age group) whereas deaths due to falls were more common in older people (38% of unintentional injury deaths at ages 60–69 years and 63% at ages 70 years and older). Age-distribution of unintentional injury death proportions for the three leading causes among males and females are reported in Additional file 1: Figure S1 and Additional file 2: Figure S2, respectively.

**Figure 2:**
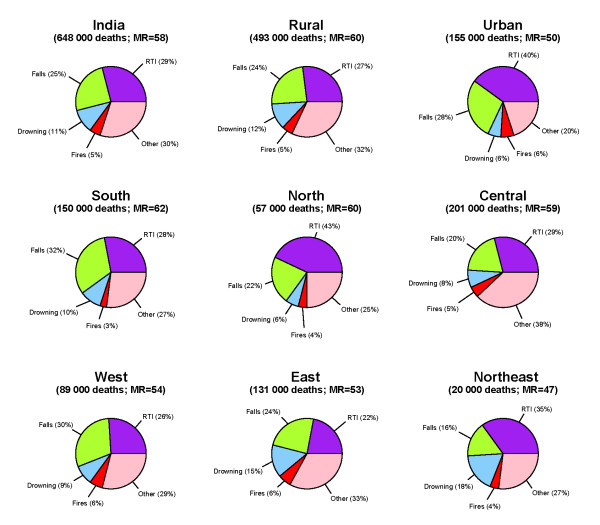
**Causes of unintentional deaths in India, for rural/urban area and six major regions, 2001–03.** Proportion of deaths by type of unintentional injury for rural/urban area and six major regions are presented for all unintentional deaths in the study sample. Number of all unintentional injury deaths and unintentional injury mortality rate (MR)/100 000 population has been reported.

The pattern of unintentional mortality in rural and urban areas was similar, however RTI constituted a higher proportion of unintentional injury deaths in urban areas (40%), while deaths due to mechanical forces (12%) and contact with venomous and plants (9%) were in higher in rural areas. The proportion of injury deaths by type also varied across the six major regions of India (Figure 2). Regional variations were also observed, with the highest unintentional injury mortality rate in South India (62 per 100 000) and the lowest in Northeast (47 per 100 000). RTI accounted for over 40% of unintentional deaths in the North, but only about 20% in the East.

**Figure 3:**
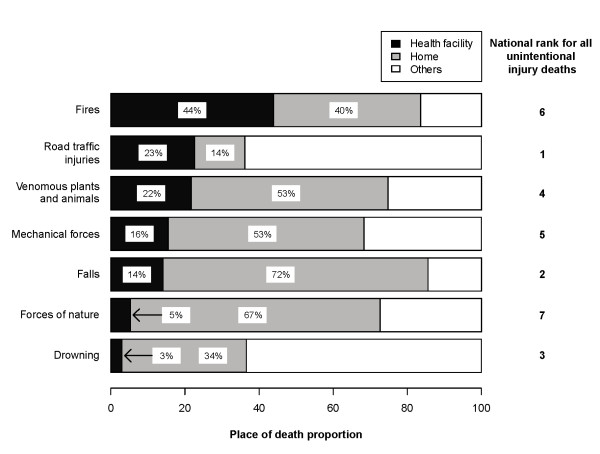
**Place of death by type of unintentional injury in India.** Proportion by place of death at home, health facility or others is presented for all unintentional deaths in the study sample. Others include places like road side, public area, on transportation and body of water. Ranking is within all unintentional injury deaths and is based on respective mortality rates for each type of injury.

Of all unintentional injury deaths, 43% occurred at home, 17% at health facilities, and 35% at other places (Figure 3). Place of death could not be determined in 5% of the deaths. About 63% of RTI deaths were recorded as occurring at other places, most often at the site of injury or on route to a health facility. Most deaths due to falls (72%) and forces of nature (67%) occurred at home. Fires were the only injury that had a high proportion (44%) of deaths in a health facility.

## Discussion

A nationally representative survey of deaths indicates that over 0.6 million persons died due to unintentional injury in India in 2005. This is twice the direct estimate of deaths from the NCRB (0.3 million) but lower than the WHO indirect estimates (0.8 million; Table 3). The underestimation in NCRB reports is likely due to reliance on police registration and thus may suffer from under-reporting by victims and families for certain types of injuries [4,5]. The WHO-Global Burden of Disease indirect estimates rely heavily on the Medically Certified Cause of Death (MCCD) (30% weight) for urban deaths and Survey of Cause of Death (SCD) (70% weight) for rural deaths, both of which rely on utilization of health facilities.

**Table 3:** Comparison of national injury death rates, per 100 000 populations from present study and other sources

**Causes**	**Present study, 2005**	**WHO/ GBD, 2004**	**National Crime Registration Bureau (NCRB), 2005**
**Road traffic injuries (V01-V89,V99)**	16.5	18.1	10.7
**Falls (W00-W19)**	14.3	8.5	0.8
**Drowning (W65-W74)**	6.4	6.3	2.1
**Fires (X00-X09)**	3.0	13.2	1.7
Poisoning* **(X40-X49)**	0.6	7.0	1.9*
**Others unintentional injuries**^†^	6.3	22.2	2.8
**Unintentional injuries V01-X59, Y40-Y86, Y88, Y89 (Number of deaths)**	**58.0 (648 000)**	**75.5 (841 699)**	**26.7 (294 175)**

*Poisoning deaths are defined differently by NCRB and includes deaths contact with venomous plants and animals and poisoning due to other agents like chemicals, liquor.

^†^Varying codes are included are used from all three sources. Present study “Others” codes include codes V90-V98, W75-W84, X10-X19, X50-X59, W85-W99, X40-X49, Y40-Y86, Y88. The WHO “Others” codes include V90-V98, W20-W64, W75-W99, X10-X39, X50-X59, Y40-Y86, Y88,Y89. Codes for “Others” by NCRB were not defined but excluded transport accidents, drowning, explosions, falls, and fires and poisoning.

Our study, a household survey using verbal autopsy method is less vulnerable to biases which affected the estimate of cause specific mortality in earlier studies (Figures 1,2,3). Indeed, we find notable differences in the age and sex composition of unintentional injury deaths between our study and the earlier MCCD, SCD and NCRB data, as well as the indirect estimates from GBD (Additional file 3: Table S1). The MCCD sample of selected urban hospitals is not representative of deaths in urban areas and suffers from quality of physician coding and completeness problems [8-10]. There are expected differences in presentation at hospital for different injuries [24]. Compared to our study, the MCCD reports a higher proportion of deaths from RTI, fires and poisoning, but lower proportions of deaths from falls and drowning (Additional file 4: Table S2).

Similarly, the SCD was based on a sample of villages with primary health care centers from selected states, and is not representative of the rural population [8-10]. It too has limitations including incomplete coverage, poor coding of causes of death and a higher proportion of ill-defined deaths [6,8]. Compared to our study, mortality proportions reported by SCD were higher for drowning and fires but lower for falls.

Mortality from RTI, particularly among males, was highest in the economically productive age group of 15–59 years, which constitutes 58% of India’s population [18]. Deaths in this age-group are likely to cause substantial household deprivation from the loss of a key wage earner [25]. Our mortality rates are consistent with the results of several local studies in India, including showing that a marked excess in young and middle aged males [26].

Our study estimates for RTI deaths are twice those reported by the NCRB. While the NCRB reported RTI deaths in urban settings might only be modestly under-reported [3,5], no comparative data exists for rural areas where the majority (77%, 1801/2339) of the RTI deaths occurred in our study. The NCRB reports also appear to overestimate the proportion of RTI deaths for heavy vehicles occupants and underestimate those from pedestrians, showing differential reporting by types of road users [2]. Similar discrepancies between police data, vital registration data and verbal autopsy based nationally representative studies have been reported in other Asian countries like China and Thailand [27,28].

Our estimates for fire related deaths in India are one-fifth of the previous indirect estimates for India from the GBD [1]. The MCCD and SCD do not classify fire related deaths by intent [6,7] making comparison to the present study difficult. Reports on deaths by family members might well under-report fire related deaths that were homicide versus unintentional, particularly in the Indian context of dowry deaths among younger adult women [2,4]. However, the proportional mortality distribution for fire related deaths by age and sex, in the present study is not markedly different from previous data sources (Additional file 3: Table S1), suggesting this bias may be modest.

On the other hand, the MCCD and SCD facility-based estimates might over-represent fire related deaths. Indeed, we noted much higher proportion of fire related deaths in a health facility (44%) as compared to all unintentional injury deaths in a health facility (17%). Further studies are required that compile multiple sources of mortality, hospital and forensic data to quantify reliably the age and gender-specific patterns of fire related deaths. Yet, the observed high proportion of fire related deaths among young adult women remains of significant concern.

Fall and drowning deaths are less likely to be medically certified and therefore would be under represented in national estimates based on hospital/medical facility data leading to an under estimation of deaths due to drowning and fall. As noted in earlier studies in the South Asia region including Bangladesh [29,30], drowning was the leading cause of unintentional death at ages 0–4 years, causing 22 000 deaths every year with higher rates in rural than in urban areas. Drowning deaths in children younger than 5 years are higher in the eastern and north eastern regions of India, which are the delta areas for major rivers, the Ganges and the Brahmaputra [21].

Mortality rates due to falls in all age groups were found to be 1.7 fold higher than those estimated indirectly [1], but consistent with recent local studies from India [5,31-33]. While pediatric falls and related traumatic brain injuries have been studied somewhat in the South Asia Region [29], there is little literature on falls in older people [33]. Our study reports three times higher deaths among older ages of 60 years and beyond, than the MCCD [7]. With a rising aged population, falls are a significant emerging public health issue in India.

### Limitations

Our study had some limitations. Verbal autopsy methods are known to misclassify some causes of death among neonates and older age groups of 70 years and above [10,34]. Earlier comparisons of verbal autopsy to urban hospital records in India indicated a sensitivity of 85% and specificity of over 95% for injuries [35]. We caution, however, that hospital-based studies are not ideal studies as a large majority of deaths occur in India without medical consultation [36] and also because of the differences observed in age-sex composition of injury deaths recorded in health facility versus those recorded at home (Additional file 3: Table S1). Misclassification for injuries overall has been low in verbal autopsy validation studies elsewhere, with the exception of falls where some misclassification with cerebro-vascular conditions was reported [37]. We estimate the injury deaths for year 2005 using proportionate injury mortality recorded during 2001-03. We did not observe any change in proportionate mortality from 2001 to 2003, hence, assumed that proportionate mortality would not have changed in next two year also, however, that may not be the case.

The disease burden in India is undergoing a transition with the burden of both chronic conditions and injury rapidly rising. However, injury is a neglected epidemic in India and few resources are dedicated towards prevention or treatment of injuries. Our results provide reliable national and regional estimates of injury mortality which can inform the allocation of resources and development of an evidence based national and state policy for injury control. Our results suggest upward revision is needed of falls mortality and perhaps downward revision of fire related deaths. Future research should aim to formulate effective injury surveillance systems, epidemiological assessment of all outcomes of injuries, advocacy for prevention and treatment and appraisal of existing effective interventions for injury prevention and trauma care.

## Conclusions

These direct estimates of unintentional injury deaths in India (0.6 million) are lower than WHO indirect estimates (0.8 million), but double the estimates which rely on police reports (0.3 million). Importantly, they revise upward the mortality due to falls, particularly in the elderly, and revise downward mortality due to fires. Road traffic injuries, falls and drowning are the leading cause of unintentional injury deaths in India.

## Abbreviations

GBD, Global Burden of Disease; ICD, International Classification of Disease; MCCD, Medically Certified Cause of Death; NCRB, National Crime and Records Bureau; RGI, Registrar General of India; SRS, Sample Registration System; SCD, Survey of Causes of Death; WHO, World Health Organisation.

## Competing interests

The authors declare they have no competing interests.

## Authors' contributions

PJ and the academic partners in India (MDS Collaborators) planned the Million Death Study in close collaboration with the RGI. JJ, WS and PJ conducted the analyses and drafted the paper. PJ is the chief investigator and the guarantor for the study. All authors participated in interpreting the data and writing the manuscript. All authors read and approved the final manuscript.

## Financial disclosures

This work was supported by the Fogarty International Centre of the US National Institutes of Health [grant R01 TW05991–01]; Canadian Institute of Health Research [CIHR; IEG-53506]; International Research Development Centre [Grant 102172]; and Li Ka Shing Knowledge Institute at St. Michael’s Hospital, University of Toronto (CGHR support). PJ is supported by the Canada Research Chair program. JJ is supported by Endeavour Research Fellowship Programme. The funding sources had no role in study design or conduct, including data collection, analysis, and interpretation. PJ had full access to all data and final responsibility for the decision to submit for publication on behalf of all authors.

## Pre-publication history

The pre-publication history for this paper can be accessed here:

http://www.biomedcentral.com/1471-2458/12/487/prepub

## Supplementary Material

Additional file 1 **Figure S1.** Age-distribution of unintentional injury mortality for the three leading causes of injuries among males in the study population, 2001–03. The three leading causes of unintentional injuries are presented as a proportion of all unintentional deaths among males in the sample. Click here for file

Additional file 2 **Figure S2.** Age-distribution of unintentional injury mortality for the three leading causes of injuries among females in the study population, 2001–03. The three leading causes of unintentional injuries are presented as a proportion of all unintentional deaths among males in the sample. Click here for file

Additional file 3 **Table S1.** Proportions of unintentional injury and fire-related deaths by age and sex group from mortality surveys, indirect estimates and the present study. Click here for file

Additional file 4 **Table S2.** Comparison of injury proportions (%) to total deaths at all ages in rural and urban areas, from present study and other data sources. Click here for file
