# Supplementary figures and images for: Unintentional injury mortality in India, 2005: Nationally representative mortality survey of 1.1 million homes
Source: BMC Public Health. 2012 Jun 28;12:487. doi: 10.1186/1471-2458-12-487 (PMC3532420; doi:10.1186/1471-2458-12-487)

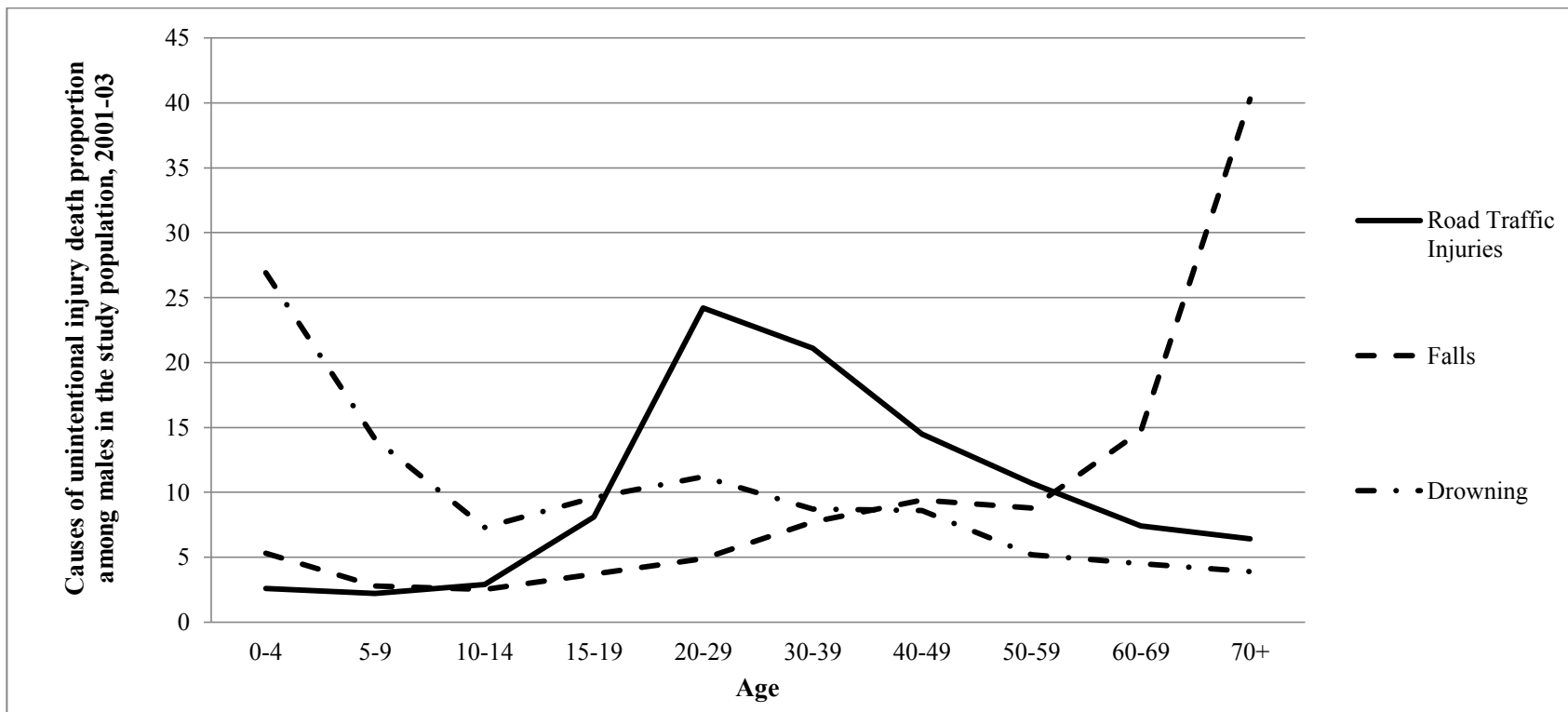

Supplement: Additional file 1 — Figure S1. Age-distribution of unintentional injury mortality for the three leading causes of injuries among males in the study population, 2001–03. The three leading causes of unintentional injuries are presented as a proportion of all unintentional deaths among males in the sample. [file 1471-2458-12-487-S1.pdf]

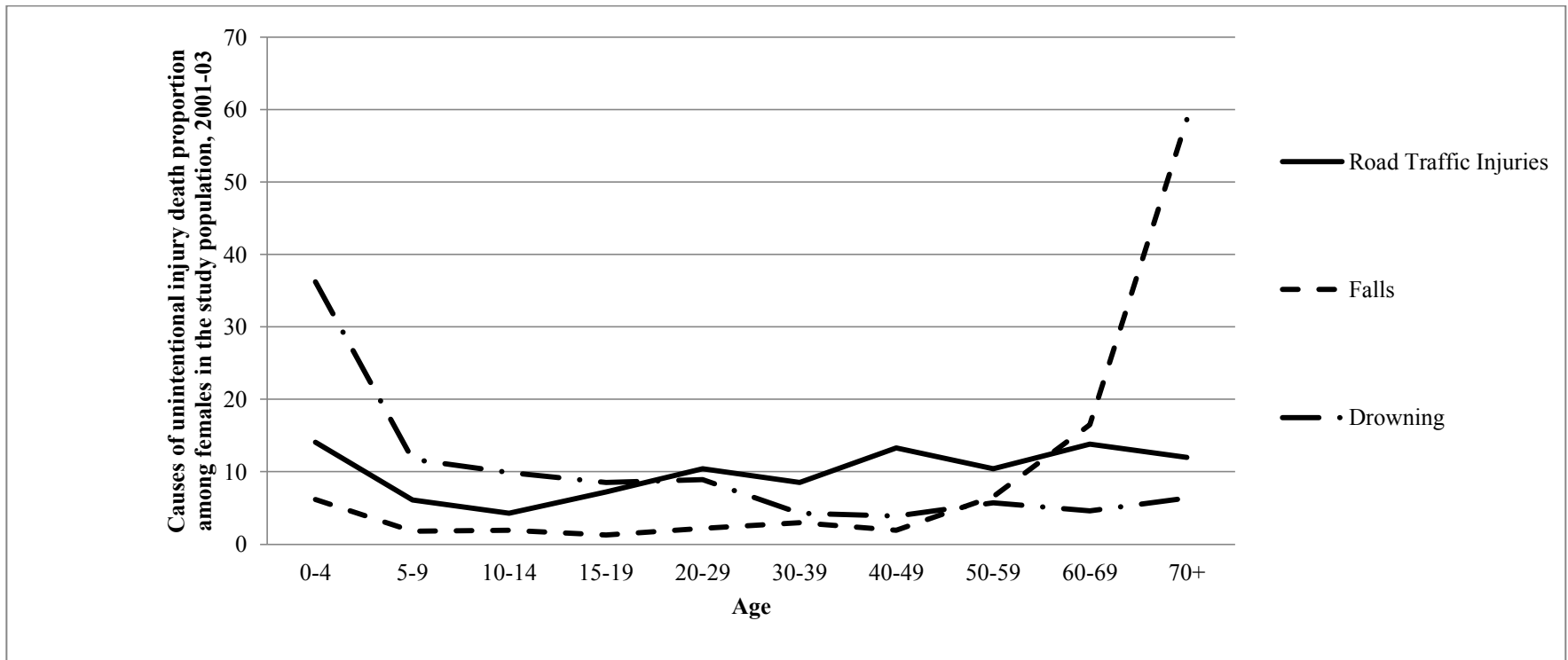

Supplement: Additional file 2 — Figure S2. Age-distribution of unintentional injury mortality for the three leading causes of injuries among females in the study population, 2001–03. The three leading causes of unintentional injuries are presented as a proportion of all unintentional deaths among males in the sample. [file 1471-2458-12-487-S2.pdf]
