# Supplementary material for: Unintentional injury mortality in India, 2005: Nationally representative mortality survey of 1.1 million homes
Source: BMC Public Health. 2012 Jun 28;12:487. doi: 10.1186/1471-2458-12-487 (PMC3532420; doi:10.1186/1471-2458-12-487)
Supplement: Additional file 3 — Table S1. Proportions of unintentional injury and fire-related deaths by age and sex group from mortality surveys, indirect estimates and the present study. [file 1471-2458-12-487-S3.doc]

| **Additional tables**  **Table S1:** **Comparison of injury proportions (%) to total deaths at all ages in rural and urban areas, from present study and other data sources** | | | | | | | |
| --- | --- | --- | --- | --- | --- | --- | --- |
| **Unintentional injury type** (ICD-10 code) | **Rural** | | | **Urban** | | | |
|  | **Survey of cause of death** | **Present study** | | **Medically certified cause of death** | **Present study** | | |
|  | **1997-98** | **2001-03** | | **2001-03** | **2001-03** | | |
| **Transport accidents** (V01-V99) | 28 | | 27 | 42 | 40 | | |
| **Falls** (W00-W19) | 7 | | 24 | 3 | 28 | | |
| **Drowning** (W65-W74) | 15 | | 12 | 3 | 6 | | |
| **Contact with venomous animals** (X20-X27) | 10 | | 9 | 2 | 2 | | |
| **Fires** (X00-X09)* | 14 | | 5 | 19 | 6 | | |
| **Other unintentional injuries**^†^(W20-W64, W75-W99, X10-X19, X30-X59, Y40-Y86, Y88) | 26 | | 23 | 31 | 18 | | |
| **Unintentional injuries** (V01-X59, Y40-Y86, Y88, Y89) | **8** | | **7** | **10** | |  | **7** |
|  | | | | | | | |
|  | | | | | | | |
