# Supplementary material for: Unintentional injury mortality in India, 2005: Nationally representative mortality survey of 1.1 million homes
Source: BMC Public Health. 2012 Jun 28;12:487. doi: 10.1186/1471-2458-12-487 (PMC3532420; doi:10.1186/1471-2458-12-487)
Supplement: Additional file 4 — Table S2. Comparison of injury proportions (%) to total deaths at all ages in rural and urban areas, from present study and other data sources. [file 1471-2458-12-487-S4.doc]

**TableS2: Proportions of unintentional injury and fire-related deaths by age and sex group from mortality surveys, indirect estimates and the present study.**

| **Data Sources** | | **Proportion of all unintentional injury deaths (%)** | | | | **Proportion of all fire related injury deaths (%)** | | | |
| --- | --- | --- | --- | --- | --- | --- | --- | --- | --- |
|  |  | **Age in years*** | | | | | | | |
|  |  | 0-14 | 15-34 | 35-59 | Above 60 years | 0-14 | 15-34 | 35-59 | Above 60 years |
| **Rural** | | | | | | | | | |
| Survey of Cause of Death,1998 | |  |  |  |  |  |  |  |  |
|  | Male | 9 | 24 | 16 | 12 | 5 | 13 | 8 | 3 |
|  | Female | 7 | 17 | 7 | 8 | 7 | 46 | 11 | 7 |
| Present study, 2001- 03 | |  |  |  |  |  |  |  |  |
|  | Male | 12 | 19 | 18 | 16 | 10 | 9 | 4 | 6 |
|  | Female | 8 | 7 | 6 | 14 | 9 | 38 | 14 | 10 |
| **Urban** | | | | | | | | | |
| Medically Certified Cause of Death, 2003 | |  |  |  |  |  |  |  |  |
|  | Male | 4 | 27 | 30 | 6 | 4 | 14 | 13 | 2 |
|  | Female | 3 | 17 | 10 | 3 | 4 | 45 | 14 | 3 |
| Present study, 2001-03 | |  |  |  |  |  |  |  |  |
|  | Male | 5 | 22 | 22 | 17 | 0 | 11 | 15 | 5 |
|  | Female | 5 | 6 | 6 | 17 | 4 | 39 | 14 | 11 |
| **All India** | |  | | | |  |  |  |  |
| National Crime Research Bureau, 2005 | |  |  |  |  |  |  |  |  |
|  | Male | 5 | 20 | 44 | 7 | 3 | 9 | 17 | 2 |
|  | Female | 2 | 8 | 11 | 3 | 5 | 31 | 28 | 5 |
| Global Burden of Disease, 2004 | |  |  |  |  |  |  |  |  |
|  | Male | 8 | 13 | 27 | 12 | 5 | 9 | 15 | 3 |
|  | Female | 8 | 10 | 13 | 9 | 9 | 27 | 24 | 8 |
| Present Study, 2001-03 | |  |  |  |  |  |  |  |  |
|  | Male | 11 | 20 | 18 | 15 | 8 | 9 | 6 | 6 |
|  | Female | 8 | 7 | 6 | 14 | 8 | 39 | 14 | 10 |

^†^ SCD and MCCD fire death codes include all injuries, while GBD and NCRB reports unintentional fire related deaths only.

The classification and available codes between sources varied.

*For MCCD the age range is 35-64 years and 65 years and above as proportional mortality is reported for 15 years intervals.

Similarly, age range is 30-59 years for NCRB and GBD.
